# Nasal resistance and OSA

**DOI:** 10.1016/S1808-8694(15)31035-1

**Published:** 2015-10-19

**Authors:** Murat Enoz, Mustafa Sitki Gozeler

**Affiliations:** a Dr. (Specialist); b Dr. (Instructor in Department of Otolaryngology, Head & Neck Surgery, Istanbul University, School of Medicine, Turkey) Department of Otolaryngology, Head & Neck Surgery, Istanbul University, School of Medicine, Turkey.

**Keywords:** nasal resistance, osa, snoring

## Abstract

Letters regarding the paper “Epidemiological study of the structural alterations present in the nasal cavity associated to the obstructive sleep apnea syndrome (OSAS)”, published on the Brazilian Journal of Otorhinolaryngology 72 (4) July/August 2006. Paper available at: http://www.rborl.org.br/conteudo/acervo/acervo_english.asp?id=3258

## LETTER

Dear Editor,

Most studies of the pathophysiology of obstructive sleep apnea syndrome (OSA) have emphasized anatomical abnormalities of the oropharyngeal and hypopharyngeal airways. Yet the nose and its impact on snoring and OSAS have not been completely ignored.

Dr. Neto^(1)^ and his friends emphasized that, structural alterations of the nasal cavity have high incidence in patients with OSA. These structural alterations such as septal deviation, conchal hypertrophy or others lead to obstruction of natural air flow and consequently increased nasal resistance.

Some studies reported that, nasal resistance has no impact on the pathogenesis of OSA. Thus, both snoring and sleep apnea are probably caused by other factors, such as restrictive processes in the pharyngeal area, rather than increased nasal resistance^2^, ^3^, ^4^.

Metes et al. did not find any effect on snoring, apneas, hypopneas or oxygen saturation in a small sample of patients, despite a reduction in nasal resistance^(5)^.

Nasal surgery for OSA usually has a very positive effect on improving the quality of life and CPAP tolerance in OSA patients^3^, ^5^.
